# A microneedle patch for measles and rubella vaccination: a game changer for achieving elimination

**DOI:** 10.1016/j.coviro.2020.05.005

**Published:** 2020-04

**Authors:** Mark R Prausnitz, James L Goodson, Paul A Rota, Walter A Orenstein

**Affiliations:** 1 School of Chemical & Biomolecular Engineering, Georgia Institute of Technology, 311 Ferst Drive, Atlanta, GA 30332, USA; 2 Centers for Disease Control and Prevention, 1600 Clifton Road, Atlanta, GA 30329, USA; 3 Emory Vaccine Center, Emory University, Atlanta, GA 30322, USA

## Abstract

While morbidity and mortality associated with measles and rubella (MR) have dramatically decreased, there are still >100 000 estimated deaths due to measles and an estimated 100 000 infants born with congenital rubella syndrome annually. Given highly effective MR vaccines, the primary barrier to global elimination of these diseases is low vaccination coverage, especially among the most underserved populations in resource-limited settings. In contrast to conventional MR vaccination by hypodermic injection, microneedle patches are being developed to enable MR vaccination by minimally trained personnel. Simplified supply chain, reduced need for cold chain storage, elimination of vaccine reconstitution, no sharps waste, reduced vaccine wastage, and reduced total system cost of vaccination are advantages of this approach. Preclinical work to develop a MR vaccine patch has proceeded through successful immunization studies in rodents and non-human primates. On-going programs seek to make MR vaccine patches available to support MR elimination efforts around the world.

**Current Opinion in Virology** 2020, **41**:68–76

This review comes from a themed issue on **Special section: measles**

Edited by **Rik L de Swart** and **Makoto Takeda**

For a complete overview see the Issue and the Editorial

Available online 1st July 2020

**https://doi.org/10.1016/j.coviro.2020.05.005**

1879-6257/© 2020 The Author(s). Published by Elsevier B.V. This is an open access article under the CC BY license (http://creativecommons.org/licenses/by/4.0/).

## Introduction

For more than a millennium, the measles virus caused human devastation and death, infecting nearly everyone [1]. Measles and rubella are vaccine-preventable and eradicable diseases; however, measles remains one of the leading causes of childhood morbidity and mortality, and rubella is the leading cause of vaccine-preventable birth defects [2^•^,3^•^]. Measles virus infection causes severe viremia and lymphopenia; common measles complications in children include otitis media, pneumonia, and diarrhea. Encephalitis, which can result in permanent brain damage, occurs in approximately 1–4 measles cases per 1000 measles cases [4, 5, 6, 7]. Measles case-fatality ratios vary widely from <0.01 to >5%, depending on comorbidities, nutritional status, and access to health care [8], and can be as high as 30% in children during major humanitarian crises [9]. Measles virus infection also causes immunosuppression that can last for months to years, diminishing preexisting antibodies against other pathogens [1,10, 11, 12, 13, 14]. Rubella virus infection generally causes a mild illness; however, when the infection occurs during pregnancy, especially in the first trimester, miscarriage, fetal death, stillbirth, or a constellation of birth defects known as congenital rubella syndrome (CRS) can occur [2^•^].

Measles and rubella viruses are airborne pathogens that are transmitted person-to-person primarily by inhalation of aerosolized respiratory droplets from an infectious person [15,16]. Dendritic cells in the respiratory tract are primary targets of measles virus in the pathogenesis of infection; measles virus can also directly infect alveolar macrophages in the lung [17,18]. Measles virus-infected dendritic cells and lymphocytes migrate throughout the body and eventually enter the subepithelial cell layers of the respiratory tract, damaging the upper respiratory tract epithelium; then the virus is shed through coughing and sneezing [19].

Measles is one of the most highly contagious infectious pathogens known; rubella is less transmissible than measles [15,20]. The basic reproduction number (R_0_), which is the measure of transmissibility that determines herd immunity thresholds and vaccination coverage levels needed to interrupt transmission, is 12–18 for measles and is 6–7 for rubella. These R_0_ values correspond to population herd immunity thresholds of 92%–94% for measles and 83%–85% for rubella [21,22]. These thresholds and the effectiveness of MR vaccine drive vaccination coverage targets for the MR elimination strategies that include providing two doses of MR vaccine and targeting ≥95% two-dose coverage among all populations around the world, with the goal of achieving and maintaining MR elimination [1,23,24^••^].

## The problem

Measles elimination goals have been established in all six WHO regions, although currently none are being met, and global efforts to achieve these goals have encountered recent setbacks [3^•^,^25^]. The World Health Assembly (WHA) endorsed the Global Vaccine Action Plan (GVAP) in 2012, with the objective to achieve measles and rubella elimination in five of the six WHO regions by 2020, and all six WHO regions have established measles elimination goals; however, resource and political commitments needed to achieve these goals have fallen short [3^•^,^26^, ^27^, ^28^]. After reaching a historic low of 132 137 reported measles cases worldwide in 2016, measles cases increased 460% in 2019 to 740 134; and estimated global measles deaths have increased from a historic low of <100,000 in 2016 to >140 000 in 2018 [3^•^]. Much of these increases were the result of large outbreaks in both industrialized and developing countries including Brazil, Chad, the Democratic Republic of the Congo, Iraq, Israel, Lebanon, Madagascar, New Zealand, Nigeria, Pakistan, the Philippines, Somalia, Sudan, Thailand, Ukraine, the United Kingdom, Venezuela, and Yemen. The measles virus importations into the United States led to 1,282 reported cases in 2019, the highest since 1992.

In many countries, vaccination coverage levels hit a plateau or even started to decrease owing to insufficient investments by governments and partners, barriers to access to vaccination, and in some settings, misinformation and concerns about safety, which lead to loss of trust and confidence in vaccines [3^•^,^28^,^29^]. To reverse the current trends, it is imperative that the global health community urgently intensify efforts and make resource commitments to fully implement the evidence-based elimination strategies, including support for new research and innovations [30]. Measles and rubella elimination research priorities for elimination have been identified, including developing new and innovative tools for increasing vaccination coverage [31,32]. Moreover, a global guidance document, the ‘Immunization Agenda 2030: A Global Strategy to Leave No One Behind’ (IA2030), that builds on lessons learned and progress made toward the GVAP goals will be approved by the WHA in May 2020 [33]. The IA2030 document includes research and innovation as a core strategic priority and identifies measles as a ‘tracer’ for improving immunization services and strengthening primary health care systems [33].

## The solution: increase MR vaccination coverage

Conventional MR vaccination efforts in many countries have failed to achieve sufficiently high coverage to achieve elimination. The need for expert health care workers to administer the vaccine, the lack of cold-chain infrastructure to reach remote locations, and costs and complexities associated with reconstitution of lyophilized vaccine, sharps waste disposal, vaccine wastage with use of multi-dose vials, and vaccination hesitancy are among the causes of low coverage [34,35,36^••^]. These problems become especially acute in conflict situations and humanitarian crises and during mass vaccination campaigns, but also present difficulties during routine vaccination as well.

**Figure 1:**
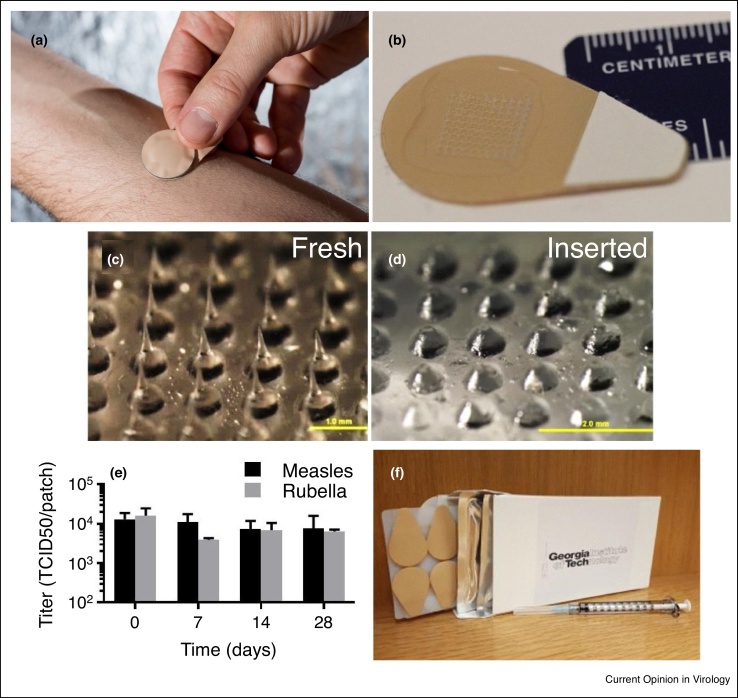
Microneedle patches for measles and rubella vaccination. Microneedle patch **(a)** being applied to the skin and **(b)** shown close-up. Magnified view of microneedle array **(c)** before and **(d)** after application and dissolution into the skin. Microneedles are 700 μm long. **(e)** Stability of measles and rubella vaccine in microneedle patches stored at 40°C for up to 28 days without significant loss of vaccine activity. **(f)** Box of 50 microneedle patches shown with a 1 mL needle and syringe. Reproduced with permission from (a) Rob Felt, Georgia Tech and (b–f) Ref. [36^••^].

To overcome the limitations of the conventional MR vaccination, microneedle (MN) patches are being developed to enable a simplified vaccination that can potentially increase MR vaccination coverage (Table 1) [37, 38, 39, 40, 41]. MN patches under advanced development for MR vaccination are skin patches measuring a few square centimeters in size that contain an array of micron-scale needles that administer vaccine (Figure 1a,b). Each MN measuring hundreds of microns in length is made of safe, water-soluble materials, and encapsulates the MR vaccine (Figure 1c). Upon insertion into the skin, the MNs dissolve, releasing the vaccine into the body (Figure 1d). An alternate design involves MNs made of non-water-soluble materials, coated with vaccine, that dissolves off in the skin.

**Table 1:** Key attributes of microneedle patch for measles and rubella vaccination and of the conventional hypodermic injection methods

Attribute	Microneedle patch	Hypodermic injection
Vaccination expertise	Minimally trained personnel	Trained health care personnel
Thermostability	Controlled temperature chain possible	Strict continuous cold chain required
Reconstitution	No reconstitution needed	Reconstitution required
Sharps waste	No sharps waste generated	Sharps waste generated
Vaccine wastage	Single-dose presentation minimizes wastage	Multi-dose presentation and limited stability after reconstitution cause vaccine wastage
Total system cost	Vaccine patch is more costly; delivery costs are reduced	Vaccine is inexpensive; most cost is in delivery
Pain	Painless vaccination	Painful vaccination

According to Pubmed (https://pubmed.ncbi.nlm.nih.gov), there have been more than 1000 papers published since the publication of the first paper on MNs for drug delivery in 1998 [42]. MN patches have been developed for influenza vaccination and studied in successful Phase 1 clinical trials showing robust immune responses, good safety profiles and relatively mild skin reactogenicity [43^••^,^44^]. MN patches have also been studied in clinical trials (79 studies listed at clinicaltrials.gov—https://clinicaltrials.gov/) for the delivery of parathyroid hormone to treat osteoporosis, zolmitriptan to treat migraine, and others to treat various ailments [45, 46, 47].

### Current research on MR vaccine patch

Measles vaccination via the skin using methods other than MN patches has had limited success, as indicated by inconsistent results in clinical trials that assessed intradermal delivery of the measles vaccine. The efficient and reliable delivery of the vaccine may be to blame. In these studies, the doses administered ranged from as low as 40 TCID_50_ to a full human dose (1000 TCID_50_); a variety of delivery methods were used including intradermal injection with needle and syringe, jet injection, and scarification by bifurcated needle [48].

**Figure 2:**
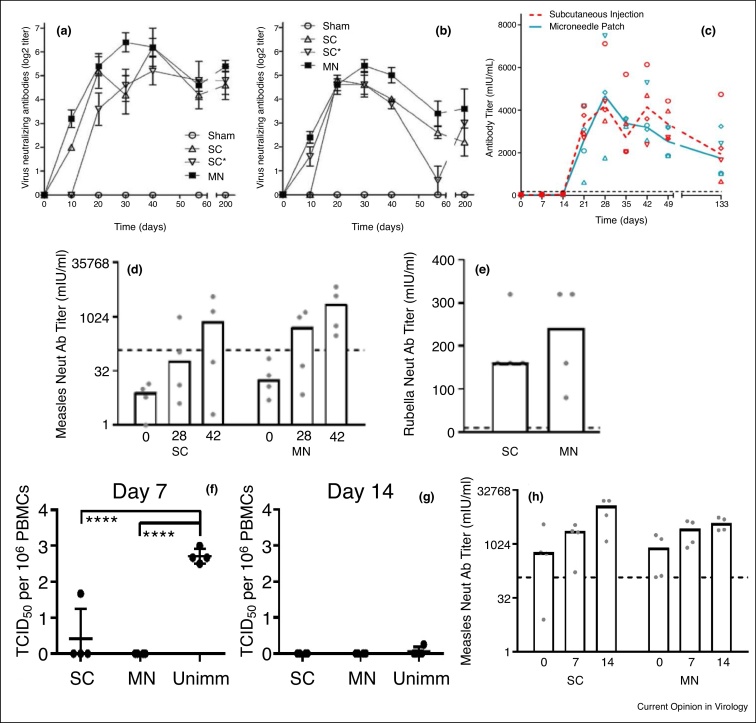
Immune responses to measles and rubella vaccination by MN patch. Virus-neutralizing antibody titers after measles vaccination by MN patch or SC injection at **(a)** a full human dose and **(b)** 20% of a full human dose of vaccine in cotton rats and **(c)** a full human dose in rhesus macaques. Virus-neutralizing antibody titers and measles and rubella vaccination by MN patch or SC injection in infant rhesus macaques: **(d)** anti-measles titers, **(e)** anti-rubella titers. Horizontal dashed lines indicate protectives titers for measles and rubella. Measles virus titers in PBMCs from infant rhesus macaques measured **(f)** 7 days and **(g)** 14 days after challenge with wild-type measles virus ∼7 months after measles and rubella vaccination by MN patch or SC injection. **(h)** Virus-neutralizing antibody titers are shown 0, 7, and 14 days post-challenge. The horizontal dashed line indicates protective titer for measles. SC, subcutaneous injection. SC*, Subcutaneous injection of reconstituted microneedle patch. MN, microneedle patch. Unimm, unimmunized. PBMC, peripheral blood mononuclear cell. Reproduced with permission from (a) Ref. [49^•^], (b) Ref. [35], (c–h) Ref. [36^••^].

The recent development of MN patches for vaccination now provides a consistent, safe, and straightforward way to deliver vaccines to the skin, which may enable intradermal MR vaccination to be successful. Initial studies, using a metal MN array to deliver measles vaccine to cotton rats, showed that neutralization titers achieved after vaccination with the MN patch were equivalent to titers measured following subcutaneous (SC) injection of either fresh vaccine or vaccine reconstituted from a MN patch (Figure 2a,b) [49^•^]. In a follow-up study, the measles vaccine was delivered to rhesus macaques at a dose of ∼3000 TCID_50_ using a dissolving MN patch and compared to vaccination by SC injection. All of the rhesus macaques had protective levels of neutralizing antibodies (>120 mIU/mL) following vaccination by either method, and the titers measured for the MN patch group were equivalent to those of the SC group (Figure 2c) [35]. This study reported that >90% of the measles vaccine in the MNs was delivered to the skin, indicating an efficient delivery process.

After the success of measles vaccination by MN patch, the immunogenicity of a dissolving MN patch delivering both measles and rubella vaccine was evaluated in infant rhesus macaques [36^••^]. Protective titers of measles neutralizing antibodies were detected in 100% of macaques in the MN patch group and in 75% of macaques in the SC injection group (Figure 2d). Neutralizing antibody titers to rubella were also in excess of the protective level of 10 IU/mL for both groups (Figure 2e). All macaques in the MN patch vaccination group were protected from the challenge with wild-type measles virus, while 75% were protected in the SC immunization group (Figure 2f,g). The titer of measles neutralizing antibodies increased seven days after the challenge in both groups (Figure 2h). The results showed that that MR vaccination by MN patch generated protective titers of neutralizing antibodies to both measles and rubella in infant rhesus macaques.

MN patches have been used to deliver several inactivated or subunit vaccines. The results described above are the first report that a MN patch could be used to successfully deliver two live-attenuated vaccines, and do so simultaneously. In this way, the vaccines were delivered into the skin by the MN patch, automatically reconstituted in the skin and replicated to the extent that a protective immune response was generated. Given the robust immune response following vaccination by MN patch, we assume that vaccine replication occurred in antigen-presenting cells such as skin resident dendritic cells and macrophages. However, additional studies are needed to identify the target cells for replication of measles and rubella vaccine following intradermal delivery [50].

## Critical attributes of MN patches

The critical attribute of MN patches is their simplicity of administration. After removal from packaging, the MN patch is pressed to the skin and left in place while the vaccine dissolves into the skin. After a few minutes, the patch is removed from the skin and discarded. This can be done by people with no specialized training; only a brief instruction is required [43^••^,^51^]. While some MN patches require administration with an applicator, other MN patches can simply be pressed to the skin by thumb. In this way, MN patches can be used in mass vaccination campaigns by minimally trained workers, administered in remote locations by non-health care personnel, or even self-administered, if appropriate. Because of their tiny size, MN patches do not hurt when applied to the skin [52,53], and they do not cause the fear and anxiety often associated with injection using hypodermic needle and syringe [54,55]. This advantage of MN patches should lead to greater vaccine acceptability.

Since MN patches are dry formulations that automatically reconstitute themselves upon penetration into the skin where they dissolve in dermal interstitial fluid, the time, cost, expertise and risk of sometimes-fatal errors associated with vaccine reconstitution are avoided [56]. Reconstitution errors and lack of adherence to strict vaccine storage and handling requirements have led to adverse events and deaths. An MR vaccine MN patch would put an end to the horrific events that have occurred in several countries, where human error during the reconstitution step of mixing diluent with the dry lyophilized powder MR vaccine has led to tragic deaths and subsequent erosion of confidence in the vaccine and vaccinators [57,58].

After MNs have dissolved in the skin, there are no sharps remaining and the used patch can be disposed of as non-sharps waste. Coated MNs that do not dissolve require disposal as sharps waste, possibly by packaging that prevents contact with the MNs after use. Sharps-free waste not only saves disposal costs, but also prevents the dangers of needle-stick injury and needle re-use [58,59]. Vaccine wastage can also be dramatically reduced using MN patches. This is because MN patches have a single-dose presentation, which avoids the need to discard unused vaccine in multi-dose vaccine vials and can reduce vaccine wastage due to incorrect storage (e.g. outside the cold chain) due to MN patch thermostability [60,61].

Storage, distribution and disposal is simplified by MN patch vaccination. MN patches are so much smaller than the needles, syringes, vaccine vials, reconstitution vials, reconstitution syringes and sharps disposal containers needed currently for MR vaccination. MN patch packaged volume is as small as 5 cm^3^ and 10 g per dose, which means that transportation to remote locations is much easier. The supply chain can also be simplified because the dry formulation of MN patches is thermostable: studies have shown that MN patch formulations containing ∼10 000 TCID_50_ MR vaccine to be stable for at least one month at 40°C (Figure 1e) and of influenza vaccine to be stable for at least one year at 40°C [36^••^,43^••^]. This thermostability can reduce the need for a cold chain for transport and storage, or at least reduce the need for refrigeration, thereby allowing the MR vaccine MN patch to be suitable for Controlled Temperature Chain licensure [62].

MN patch vaccines may be cost-competitive with conventional vaccines, especially when the analysis accounts for absence of a costly applicator, the cost-savings associated with minimally trained personnel, thermostability, reduced vaccine wastage, no sharps waste and no reconstitution [63].

### MN patch for MR vaccination to achieve high coverage and equity

An MR vaccine MN patch is widely recognized as a potential game-changer for efforts to increase vaccination coverage and equity, could enhance implementation of MR elimination strategies and rapidly advance progress toward elimination goals. Achieving MR elimination by reaching vaccination coverage and equity targets depends on strong systems for routine immunizations service delivery, to increase MCV coverage with a first dose during infancy and a second dose given beyond the first year of life.

The single-dose packaging of an MR vaccine MN patch could contribute to increasing routine coverage by overcoming the missed opportunity for vaccination that occurs when vaccinators are reluctant to open multi-dose vials over concerns of vaccine wastage. In some settings, to avoid vaccine wastage, vaccinators attempt to batch unvaccinated children by sending them back home to come back another time with other children on specified vaccination days [64]; an MR vaccine MN patch would improve reliability and performance of immunizations service delivery. Outreach services would benefit from the simplified logistics of MN patch vaccination, with reduced storage and disposal requirements, easier transportation, and improved access to hard-to-reach areas, places in desperate need of increased vaccination coverage [34].

In settings with suboptimal vaccination coverage, periodic supplementary immunization activities (SIAs) are used to reach those who were missed by routine vaccination or were left without protective immunity [30]. In places where SIAs are still needed to reach high two-dose coverage, an MR vaccine MN patch would allow for vaccination by minimally trained personnel and make vaccination campaigns possible, which are key strategies to reach vaccination coverage targets for other elimination and eradication programs [35,36^••^]. MR vaccine MN patch use would simplify logistics to facilitate outbreak responses, improve vaccination in conflict zones, and address other humanitarian needs [65].

MN patches could help immunizations service delivery where measles-rubella combined vaccine is given, primarily in developing countries, as well as in industrialized nations, where measles-mumps-rubella or measles-mumps-rubella-varicella vaccines could be given via MN patch [41,57,66]. In these settings, vaccination coverage should increase among those with vaccine hesitancy due to needle phobia or pain, especially for existing multiple-injection clinic visits.

### MR vaccine patch development and translation into the clinic

Stimulated by the potential for an MR vaccine patch to improve vaccination programs and the initial data demonstrating its feasibility in pre-clinical studies, the Bill & Melinda Gates Foundation issued a solicitation for proposals to develop an MR vaccine patch that meets the needs of global health in 2016. Grants were awarded to three programs: a Georgia Tech–CDC–Micron Biomedical collaboration, Vaxxas, and Vaxess Technologies. Pre-clinical development is currently underway, and the first Phase 1 clinical trials are expected to begin as soon as 2020. In addition, the WHO has established the Measles-Rubella Micro-Array Patch (MR-MAP)^5^ Working Group, which has prepared a target product profile for MR vaccine patches [34]. The Working Group is investigating possible use-cases for the MR vaccine patches as inputs to understanding the potential market and cost-of-goods, and evaluating the development and use of MN patches for MR vaccination. The Program for Appropriate Technology in Health (PATH) has established a Center of Excellence for Microarray Patch Technology that seeks to accelerate the development of MN patches to meet global public health needs. Through that center, PATH has created a Regulatory Working Group that seeks to identify critical quality attributes, develop test methods to serve as standards for regulatory filings, and assess MN patch manufacturing methods [67^•^].

## Future outlook

After an intensive investment of time, personnel and resources, global MR vaccination coverage has increased, and the associated morbidity and mortality have decreased over the last two decades. However, in recent years, measles cases and deaths have increased instead of decreasing. The barrier to achieving elimination goals is low vaccination coverage, insufficient to achieve or maintain elimination, especially in medically underserved populations, which necessitates innovation in the ways of getting vaccines to hard-to-reach populations. The MN patch, as an innovation, offers the promise of expanding the reach of vaccination programs and further reducing measles deaths and cases of congenital rubella syndrome. With multiple active development programs, and at least one expected to begin a Phase 1 clinical trial in 2020, MR vaccine patches are on their way to helping MR elimination efforts succeed.

Questions remain about the path forward for MR vaccine patches. While pre-clinical studies have shown promise, human clinical trials are needed to assess more fully the safety and immunogenicity of this approach. Decisions need to be made about clinical trial designs, regulatory bodies and path to licensure, and relationships among patch technology developers, vaccine manufacturers, program funders, healthcare payers, and the global health community. The technology development risk profile must also be evaluated to determine the optimal balance between risk, cost and speed to a final product. Because the MR vaccine patch lacks the mumps vaccine component needed in industrialized countries, it’s expected market is the developing countries. Therefore, financial model needs clarification, including the players and their roles in a public-private partnership to develop the technology. Lessons learned from this analysis could apply not only to a MR vaccine patch but could help pave the way for other vaccines administered by MN patch.

Many people and organizations in the global health community are focused on these questions and are committed to bringing forward the MR vaccine patch. There is a shared hope and expectation that it can be the game-changer that increases MR vaccination coverage, enabling the global elimination of measles and rubella.

## Conflict of interest statement

MRP is an inventor of patents licensed to companies developing microneedle-based products, is a paid advisor to companies developing microneedle-based products and is a founder/shareholder of companies developing microneedle-based products (Micron Biomedical). This potential conflict of interest has been disclosed and is managed by Georgia Institute of Technology. The other authors have no conflicts of interest to declare.

## References and recommended reading

Papers of particular interest, published within the period of review, have been highlighted as:

• of special interest
•• of outstanding interest

## Footnotes

5 Another term for microneedle patch is microarray patch, that is, MAP.
